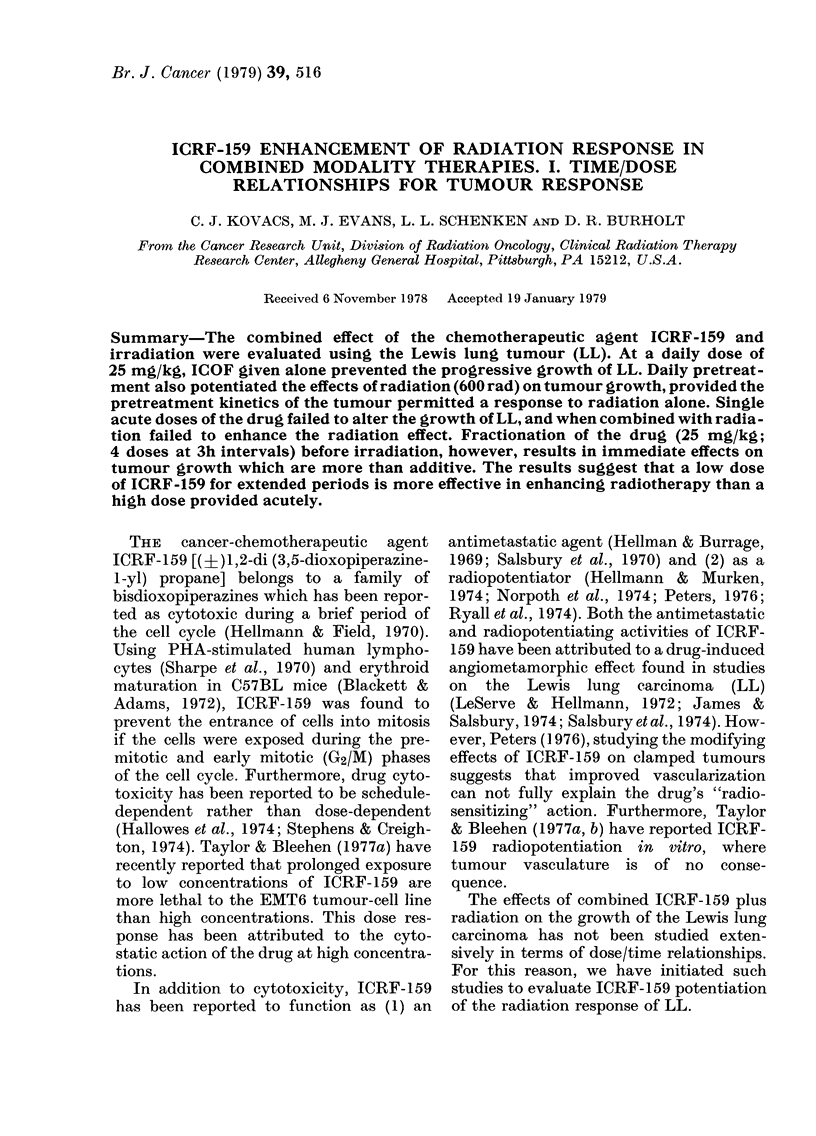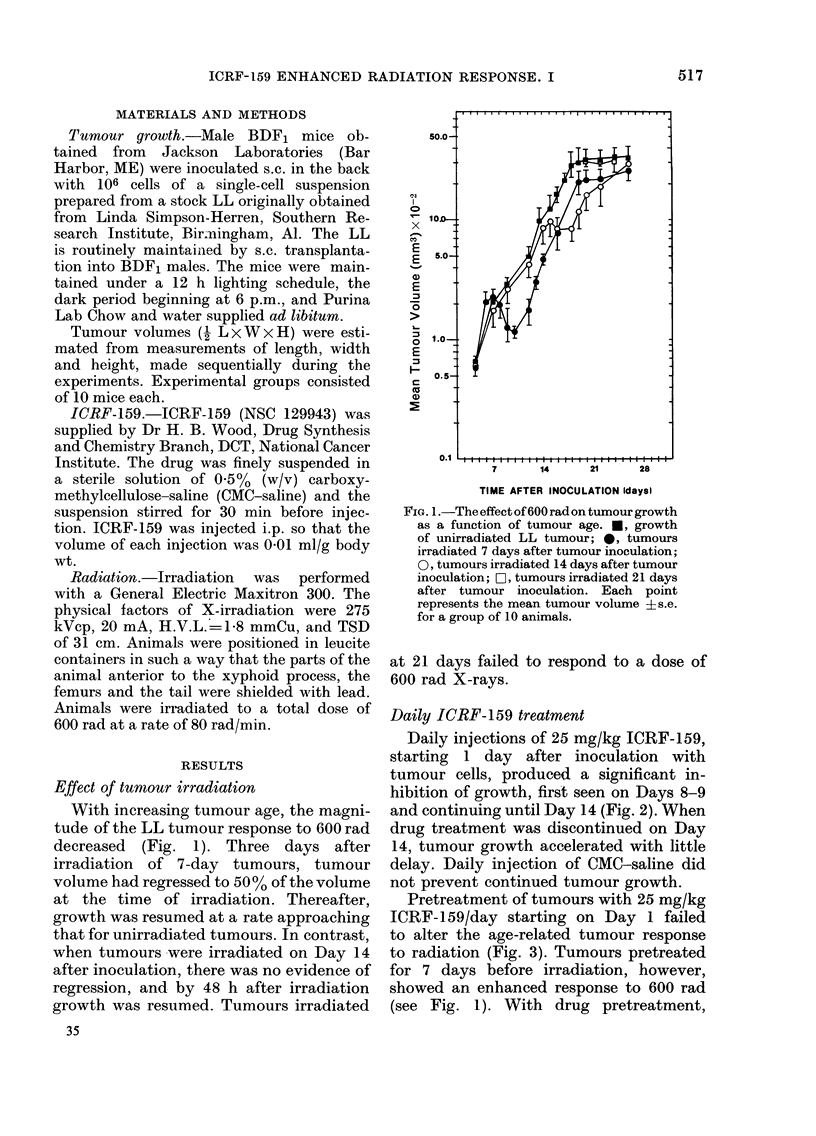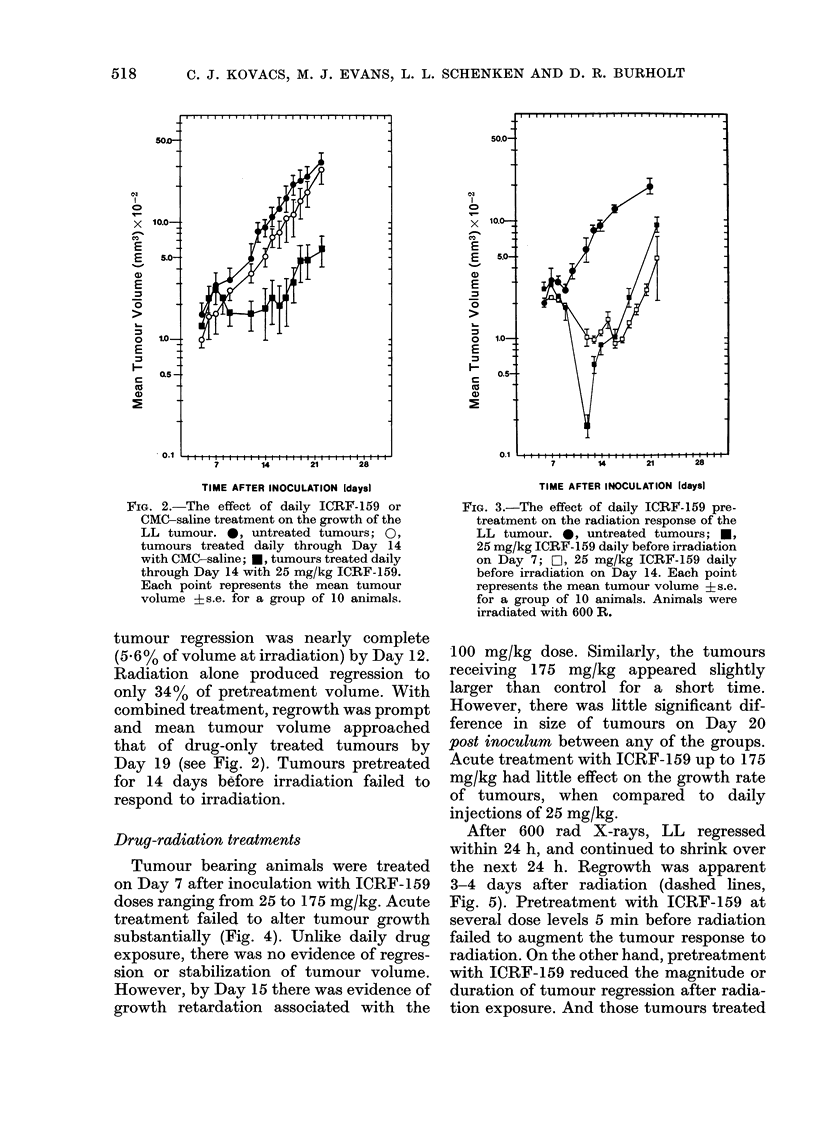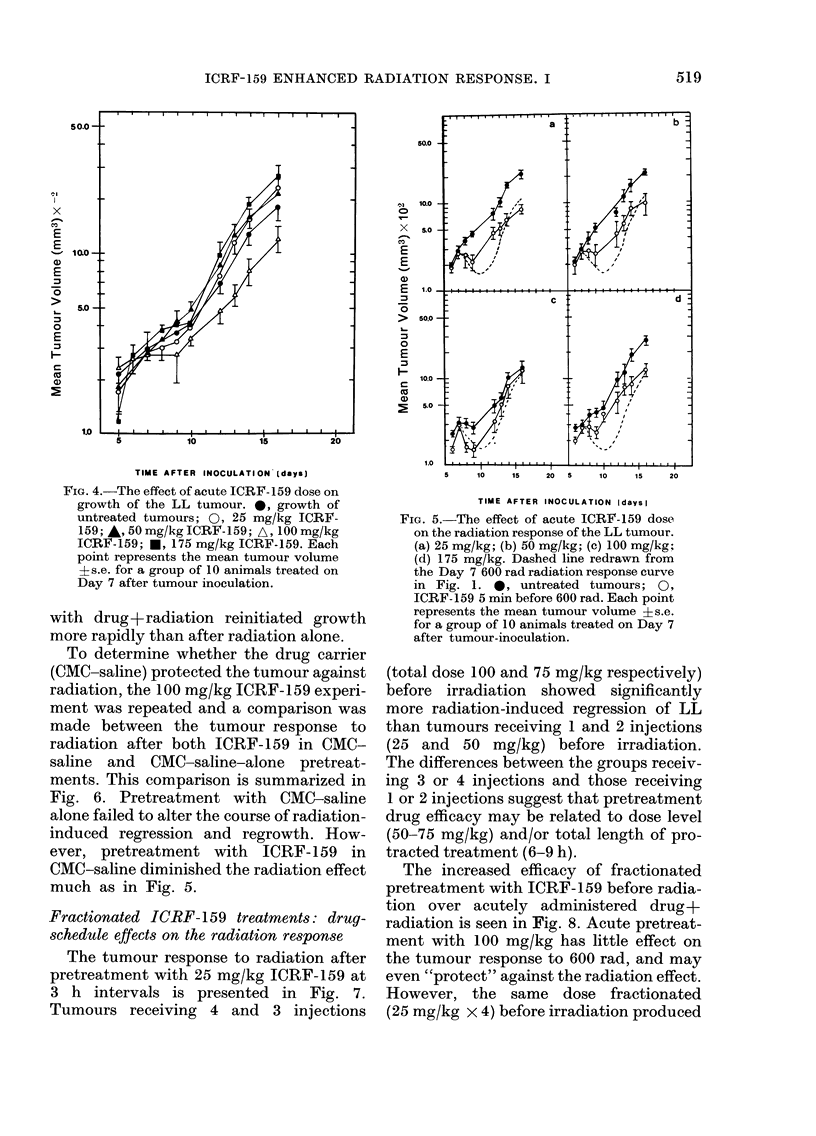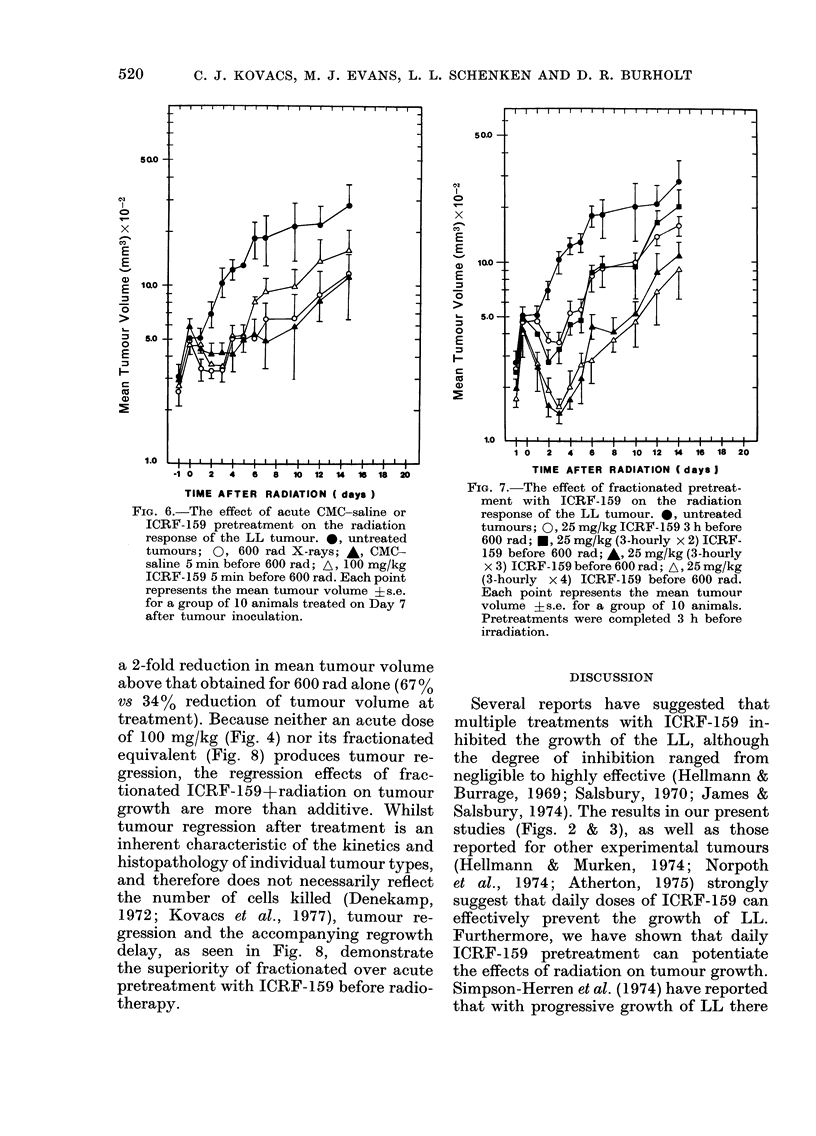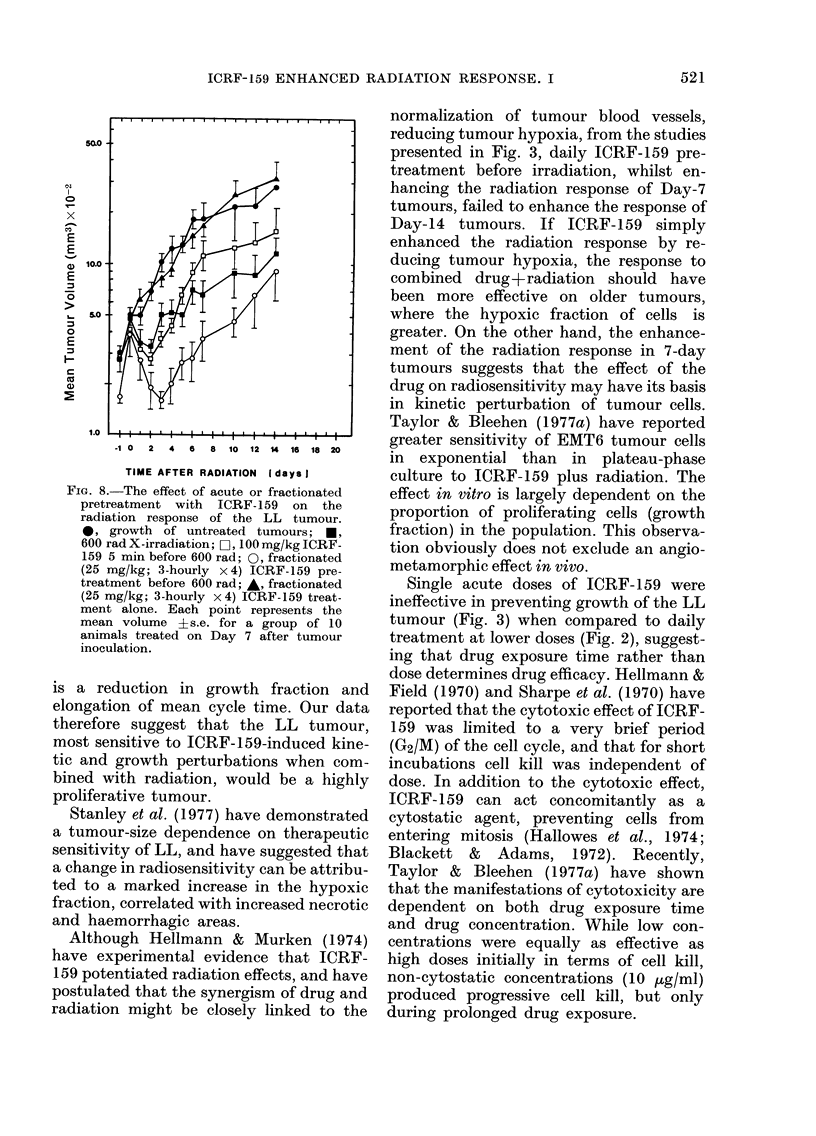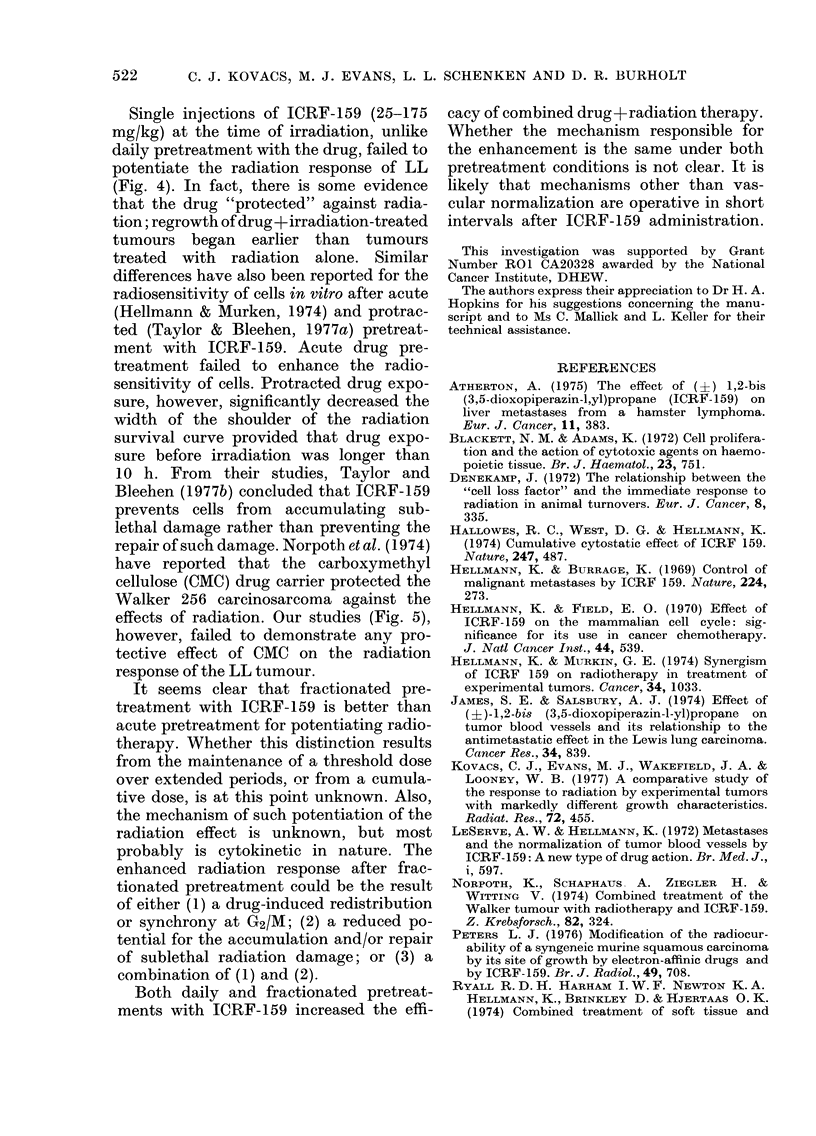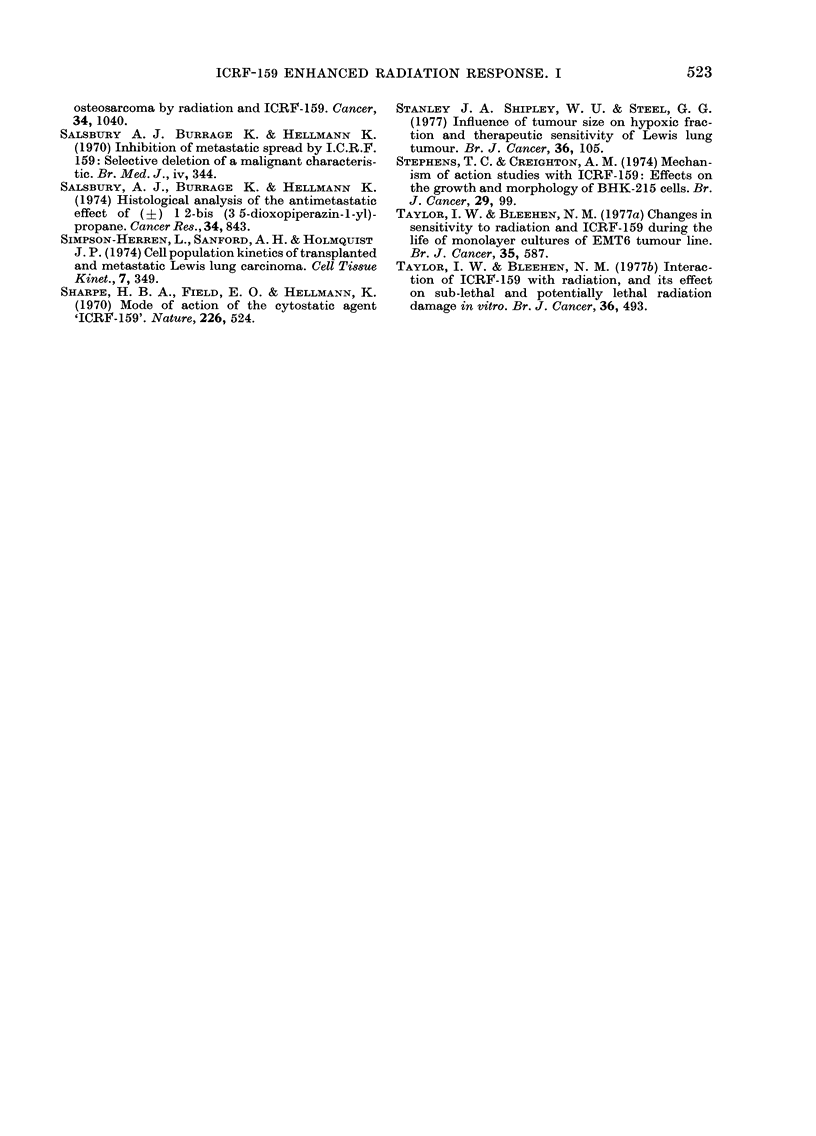# ICRF-159 enhancement of radiation response in combined modality therapies. I. Time/dose relationships for tumour response.

**DOI:** 10.1038/bjc.1979.95

**Published:** 1979-05

**Authors:** C. J. Kovacs, M. J. Evans, L. L. Schenken, D. R. Burholt

## Abstract

The combined effect of the chemotherapeutic agent ICRF-159 and irradiation were evaluated using the Lewis lung tumour (LL). At a daily dose of 25 mg/kg, ICOF given alone prevented the progressive growth of LL. Daily pretreatment also potentiated the effects of radiation (600 rad) on tumour growth, provided the pretreatment kinetics of the tumour permitted a response to radiation alone. Single acute doses of the drug failed to alter the growth of LL, and when combined with radiation failed to enhance the radiation effect. Fractionation of the drug (25 mg/kg; 4 doses at 3h intervals) before irradiation, however, results in immediate effects on tumour growth which are more than additive. The results suggest that a low dose of ICRF-159 for extended periods is more effective in enhancing radiotherapy than a high dose provided acutely.


*Br. J. Cancer* (1979) **39,** 516

### ICRF-159 ENHANCEMENT OF RADIATION RESPONSE IN COMBINED MODALITY THERAPIES. I. TIME/DOSE RELATIONSHIPS FOR TUMOUR RESPONSE

C. J. KOVACS, M. J. EVANS, L. L. SCHENKEN AND D. R. BURHOLT

*From the Cancer Research Unit, Division of Radiation Oncology, Clinical Radiation Therapy Research Center, Allegheny General Hospital, Pittsburgh, PA* 15212, *U.S.A.*

Received 6 November 1978 Accepted 19 January 1979

**Summary—The combined effect of the chemotherapeutic agent ICRF-159 and irradiation were evaluated using the Lewis lung tumour (LL). At a daily dose of 25 mg/kg, ICOF given alone prevented the progressive growth of LL. Daily pretreatment also potentiated the effects of radiation (600 rad) on tumour growth, provided the pretreatment kinetics of the tumour permitted a response to radiation alone. Single acute doses of the drug failed to alter the growth of LL, and when combined with radiation failed to enhance the radiation effect. Fractionation of the drug (25 mg/kg; 4 doses at 3h intervals) before irradiation, however, results in immediate effects on tumour growth which are more than additive. The results suggest that a low dose of ICRF-159 for extended periods is more effective in enhancing radiotherapy than a high dose provided acutely.**

THE cancer-chemotherapeutic agent ICRF-159 [($\pm$)1,2-di (3,5-dioxopiperazine-1-yl) propane] belongs to a family of bisdioxopiperazines which has been reported as cytotoxic during a brief period of the cell cycle (Hellmann & Field, 1970). Using PHA-stimulated human lymphocytes (Sharpe *et al.*, 1970) and erythroid maturation in C57BL mice (Blackett & Adams, 1972), ICRF-159 was found to prevent the entrance of cells into mitosis if the cells were exposed during the premitotic and early mitotic ($G_2$/M) phases of the cell cycle. Furthermore, drug cytotoxicity has been reported to be schedule-dependent rather than dose-dependent (Hallowes *et al.*, 1974; Stephens & Creighton, 1974). Taylor & Bleehen (1977*a*) have recently reported that prolonged exposure to low concentrations of ICRF-159 are more lethal to the EMT6 tumour-cell line than high concentrations. This dose response has been attributed to the cytostatic action of the drug at high concentrations.

In addition to cytotoxicity, ICRF-159 has been reported to function as (1) an antimetastatic agent (Hellman & Burrage, 1969; Salsbury *et al.*, 1970) and (2) as a radiopotentiator (Hellmann & Murken, 1974; Norpoth *et al.*, 1974; Peters, 1976; Ryall *et al.*, 1974). Both the antimetastatic and radiopotentiating activities of ICRF-159 have been attributed to a drug-induced angiometamorphic effect found in studies on the Lewis lung carcinoma (LL) (LeServe & Hellmann, 1972; James & Salsbury, 1974; Salsbury *et al.*, 1974). However, Peters (1976), studying the modifying effects of ICRF-159 on clamped tumours suggests that improved vascularization can not fully explain the drug's "radiosensitizing" action. Furthermore, Taylor & Bleehen (1977*a, b*) have reported ICRF-159 radiopotentiation *in vitro*, where tumour vasculature is of no consequence.

The effects of combined ICRF-159 plus radiation on the growth of the Lewis lung carcinoma has not been studied extensively in terms of dose/time relationships. For this reason, we have initiated such studies to evaluate ICRF-159 potentiation of the radiation response of LL.

MATERIALS AND METHODS

*Tumour growth.*—Male $BDF_1$ mice obtained from Jackson Laboratories (Bar Harbor, ME) were inoculated s.c. in the back with $10^6$ cells of a single-cell suspension prepared from a stock LL originally obtained from Linda Simpson-Herren, Southern Research Institute, Birmingham, Al. The LL is routinely maintained by s.c. transplantation into $BDF_1$ males. The mice were maintained under a 12 h lighting schedule, the dark period beginning at 6 p.m., and Purina Lab Chow and water supplied *ad libitum*.

Tumour volumes ($\frac{1}{2}$ L×W×H) were estimated from measurements of length, width and height, made sequentially during the experiments. Experimental groups consisted of 10 mice each.

*ICRF*-159.—ICRF-159 (NSC 129943) was supplied by Dr H. B. Wood, Drug Synthesis and Chemistry Branch, DCT, National Cancer Institute. The drug was finely suspended in a sterile solution of 0·5% (w/v) carboxymethylcellulose–saline (CMC–saline) and the suspension stirred for 30 min before injection. ICRF-159 was injected i.p. so that the volume of each injection was 0·01 ml/g body wt.

*Radiation.*—Irradiation was performed with a General Electric Maxitron 300. The physical factors of X-irradiation were 275 kVcp, 20 mA, H.V.L.=1·8 mmCu, and TSD of 31 cm. Animals were positioned in leucite containers in such a way that the parts of the animal anterior to the xyphoid process, the femurs and the tail were shielded with lead. Animals were irradiated to a total dose of 600 rad at a rate of 80 rad/min.

RESULTS

*Effect of tumour irradiation*

With increasing tumour age, the magnitude of the LL tumour response to 600 rad decreased (Fig. 1). Three days after irradiation of 7-day tumours, tumour volume had regressed to 50% of the volume at the time of irradiation. Thereafter, growth was resumed at a rate approaching that for unirradiated tumours. In contrast, when tumours were irradiated on Day 14 after inoculation, there was no evidence of regression, and by 48 h after irradiation growth was resumed. Tumours irradiated

FIG. 1.—The effect of 600 rad on tumour growth as a function of tumour age. ■, growth of unirradiated LL tumour; ●, tumours irradiated 7 days after tumour inoculation; ○, tumours irradiated 14 days after tumour inoculation; □, tumours irradiated 21 days after tumour inoculation. Each point represents the mean tumour volume ±s.e. for a group of 10 animals.

at 21 days failed to respond to a dose of 600 rad X-rays.

*Daily ICRF-159 treatment*

Daily injections of 25 mg/kg ICRF-159, starting 1 day after inoculation with tumour cells, produced a significant inhibition of growth, first seen on Days 8–9 and continuing until Day 14 (Fig. 2). When drug treatment was discontinued on Day 14, tumour growth accelerated with little delay. Daily injection of CMC–saline did not prevent continued tumour growth.

Pretreatment of tumours with 25 mg/kg ICRF-159/day starting on Day 1 failed to alter the age-related tumour response to radiation (Fig. 3). Tumours pretreated for 7 days before irradiation, however, showed an enhanced response to 600 rad (see Fig. 1). With drug pretreatment,

FIG. 2.—The effect of daily ICRF-159 or CMC–saline treatment on the growth of the LL tumour. ●, untreated tumours; ○, tumours treated daily through Day 14 with CMC–saline; ■, tumours treated daily through Day 14 with 25 mg/kg ICRF-159. Each point represents the mean tumour volume ±s.e. for a group of 10 animals.

FIG. 3.—The effect of daily ICRF-159 pretreatment on the radiation response of the LL tumour. ●, untreated tumours; ■, 25 mg/kg ICRF-159 daily before irradiation on Day 7; □, 25 mg/kg ICRF-159 daily before irradiation on Day 14. Each point represents the mean tumour volume ±s.e. for a group of 10 animals. Animals were irradiated with 600 R.

tumour regression was nearly complete (5·6% of volume at irradiation) by Day 12. Radiation alone produced regression to only 34% of pretreatment volume. With combined treatment, regrowth was prompt and mean tumour volume approached that of drug-only treated tumours by Day 19 (see Fig. 2). Tumours pretreated for 14 days before irradiation failed to respond to irradiation.

*Drug-radiation treatments*

Tumour bearing animals were treated on Day 7 after inoculation with ICRF-159 doses ranging from 25 to 175 mg/kg. Acute treatment failed to alter tumour growth substantially (Fig. 4). Unlike daily drug exposure, there was no evidence of regression or stabilization of tumour volume. However, by Day 15 there was evidence of growth retardation associated with the 100 mg/kg dose. Similarly, the tumours receiving 175 mg/kg appeared slightly larger than control for a short time. However, there was little significant difference in size of tumours on Day 20 *post inoculum* between any of the groups. Acute treatment with ICRF-159 up to 175 mg/kg had little effect on the growth rate of tumours, when compared to daily injections of 25 mg/kg.

After 600 rad X-rays, LL regressed within 24 h, and continued to shrink over the next 24 h. Regrowth was apparent 3–4 days after radiation (dashed lines, Fig. 5). Pretreatment with ICRF-159 at several dose levels 5 min before radiation failed to augment the tumour response to radiation. On the other hand, pretreatment with ICRF-159 reduced the magnitude or duration of tumour regression after radiation exposure. And those tumours treated

FIG. 4.—The effect of acute ICRF-159 dose on growth of the LL tumour. ●, growth of untreated tumours; ○, 25 mg/kg ICRF-159; ▲, 50 mg/kg ICRF-159; △, 100 mg/kg ICRF-159; ■, 175 mg/kg ICRF-159. Each point represents the mean tumour volume ±s.e. for a group of 10 animals treated on Day 7 after tumour inoculation.

FIG. 5.—The effect of acute ICRF-159 dose on the radiation response of the LL tumour. (a) 25 mg/kg; (b) 50 mg/kg; (c) 100 mg/kg; (d) 175 mg/kg. Dashed line redrawn from the Day 7 600 rad radiation response curve in Fig. 1. ●, untreated tumours; ○, ICRF-159 5 min before 600 rad. Each point represents the mean tumour volume ±s.e. for a group of 10 animals treated on Day 7 after tumour-inoculation.

with drug+radiation reinitiated growth more rapidly than after radiation alone.

To determine whether the drug carrier (CMC–saline) protected the tumour against radiation, the 100 mg/kg ICRF-159 experiment was repeated and a comparison was made between the tumour response to radiation after both ICRF-159 in CMC–saline and CMC–saline–alone pretreatments. This comparison is summarized in Fig. 6. Pretreatment with CMC–saline alone failed to alter the course of radiation-induced regression and regrowth. However, pretreatment with ICRF-159 in CMC–saline diminished the radiation effect much as in Fig. 5.

*Fractionated ICRF*-159 *treatments: drug-schedule effects on the radiation response*

The tumour response to radiation after pretreatment with 25 mg/kg ICRF-159 at 3 h intervals is presented in Fig. 7. Tumours receiving 4 and 3 injections (total dose 100 and 75 mg/kg respectively) before irradiation showed significantly more radiation-induced regression of LL than tumours receiving 1 and 2 injections (25 and 50 mg/kg) before irradiation. The differences between the groups receiving 3 or 4 injections and those receiving 1 or 2 injections suggest that pretreatment drug efficacy may be related to dose level (50–75 mg/kg) and/or total length of protracted treatment (6–9 h).

The increased efficacy of fractionated pretreatment with ICRF-159 before radiation over acutely administered drug+radiation is seen in Fig. 8. Acute pretreatment with 100 mg/kg has little effect on the tumour response to 600 rad, and may even "protect" against the radiation effect. However, the same dose fractionated (25 mg/kg ×4) before irradiation produced

FIG. 6.—The effect of acute CMC–saline or ICRF-159 pretreatment on the radiation response of the LL tumour. ●, untreated tumours; ○, 600 rad X-rays; ▲, CMC–saline 5 min before 600 rad; △, 100 mg/kg ICRF-159 5 min before 600 rad. Each point represents the mean tumour volume ±s.e. for a group of 10 animals treated on Day 7 after tumour inoculation.

FIG. 7.—The effect of fractionated pretreatment with ICRF-159 on the radiation response of the LL tumour. ●, untreated tumours; ○, 25 mg/kg ICRF-159 3 h before 600 rad; ■, 25 mg/kg (3-hourly ×2) ICRF-159 before 600 rad; ▲, 25 mg/kg (3-hourly ×3) ICRF-159 before 600 rad; △, 25 mg/kg (3-hourly ×4) ICRF-159 before 600 rad. Each point represents the mean tumour volume ±s.e. for a group of 10 animals. Pretreatments were completed 3 h before irradiation.

a 2-fold reduction in mean tumour volume above that obtained for 600 rad alone (67% *vs* 34% reduction of tumour volume at treatment). Because neither an acute dose of 100 mg/kg (Fig. 4) nor its fractionated equivalent (Fig. 8) produces tumour regression, the regression effects of fractionated ICRF-159+radiation on tumour growth are more than additive. Whilst tumour regression after treatment is an inherent characteristic of the kinetics and histopathology of individual tumour types, and therefore does not necessarily reflect the number of cells killed (Denekamp, 1972; Kovacs *et al.*, 1977), tumour regression and the accompanying regrowth delay, as seen in Fig. 8, demonstrate the superiority of fractionated over acute pretreatment with ICRF-159 before radiotherapy.

### DISCUSSION

Several reports have suggested that multiple treatments with ICRF-159 inhibited the growth of the LL, although the degree of inhibition ranged from negligible to highly effective (Hellmann & Burrage, 1969; Salsbury, 1970; James & Salsbury, 1974). The results in our present studies (Figs. 2 & 3), as well as those reported for other experimental tumours (Hellmann & Murken, 1974; Norpoth *et al.*, 1974; Atherton, 1975) strongly suggest that daily doses of ICRF-159 can effectively prevent the growth of LL. Furthermore, we have shown that daily ICRF-159 pretreatment can potentiate the effects of radiation on tumour growth. Simpson-Herren *et al.* (1974) have reported that with progressive growth of LL there

FIG. 8.—The effect of acute or fractionated pretreatment with ICRF-159 on the radiation response of the LL tumour. ●, growth of untreated tumours; ■, 600 rad X-irradiation; □, 100 mg/kg ICRF-159 5 min before 600 rad; ○, fractionated (25 mg/kg; 3-hourly ×4) ICRF-159 pretreatment before 600 rad; ▲, fractionated (25 mg/kg; 3-hourly ×4) ICRF-159 treatment alone. Each point represents the mean volume ±s.e. for a group of 10 animals treated on Day 7 after tumour inoculation.

is a reduction in growth fraction and elongation of mean cycle time. Our data therefore suggest that the LL tumour, most sensitive to ICRF-159-induced kinetic and growth perturbations when combined with radiation, would be a highly proliferative tumour.

Stanley *et al.* (1977) have demonstrated a tumour-size dependence on therapeutic sensitivity of LL, and have suggested that a change in radiosensitivity can be attributed to a marked increase in the hypoxic fraction, correlated with increased necrotic and haemorrhagic areas.

Although Hellmann & Murken (1974) have experimental evidence that ICRF-159 potentiated radiation effects, and have postulated that the synergism of drug and radiation might be closely linked to the normalization of tumour blood vessels, reducing tumour hypoxia, from the studies presented in Fig. 3, daily ICRF-159 pretreatment before irradiation, whilst enhancing the radiation response of Day-7 tumours, failed to enhance the response of Day-14 tumours. If ICRF-159 simply enhanced the radiation response by reducing tumour hypoxia, the response to combined drug+radiation should have been more effective on older tumours, where the hypoxic fraction of cells is greater. On the other hand, the enhancement of the radiation response in 7-day tumours suggests that the effect of the drug on radiosensitivity may have its basis in kinetic perturbation of tumour cells. Taylor & Bleehen (1977*a*) have reported greater sensitivity of EMT6 tumour cells in exponential than in plateau-phase culture to ICRF-159 plus radiation. The effect *in vitro* is largely dependent on the proportion of proliferating cells (growth fraction) in the population. This observation obviously does not exclude an angiometamorphic effect *in vivo*.

Single acute doses of ICRF-159 were ineffective in preventing growth of the LL tumour (Fig. 3) when compared to daily treatment at lower doses (Fig. 2), suggesting that drug exposure time rather than dose determines drug efficacy. Hellmann & Field (1970) and Sharpe *et al.* (1970) have reported that the cytotoxic effect of ICRF-159 was limited to a very brief period ($G_2$/M) of the cell cycle, and that for short incubations cell kill was independent of dose. In addition to the cytotoxic effect, ICRF-159 can act concomitantly as a cytostatic agent, preventing cells from entering mitosis (Hallowes *et al.*, 1974; Blackett & Adams, 1972). Recently, Taylor & Bleehen (1977*a*) have shown that the manifestations of cytotoxicity are dependent on both drug exposure time and drug concentration. While low concentrations were equally as effective as high doses initially in terms of cell kill, non-cytostatic concentrations (10 μg/ml) produced progressive cell kill, but only during prolonged drug exposure.

Single injections of ICRF-159 (25–175 mg/kg) at the time of irradiation, unlike daily pretreatment with the drug, failed to potentiate the radiation response of LL (Fig. 4). In fact, there is some evidence that the drug "protected" against radiation; regrowth of drug+irradiation-treated tumours began earlier than tumours treated with radiation alone. Similar differences have also been reported for the radiosensitivity of cells *in vitro* after acute (Hellmann & Murken, 1974) and protracted (Taylor & Bleehen, 1977*a*) pretreatment with ICRF-159. Acute drug pretreatment failed to enhance the radiosensitivity of cells. Protracted drug exposure, however, significantly decreased the width of the shoulder of the radiation survival curve provided that drug exposure before irradiation was longer than 10 h. From their studies, Taylor and Bleehen (1977*b*) concluded that ICRF-159 prevents cells from accumulating sublethal damage rather than preventing the repair of such damage. Norpoth *et al.* (1974) have reported that the carboxymethyl cellulose (CMC) drug carrier protected the Walker 256 carcinosarcoma against the effects of radiation. Our studies (Fig. 5), however, failed to demonstrate any protective effect of CMC on the radiation response of the LL tumour.

It seems clear that fractionated pretreatment with ICRF-159 is better than acute pretreatment for potentiating radiotherapy. Whether this distinction results from the maintenance of a threshold dose over extended periods, or from a cumulative dose, is at this point unknown. Also, the mechanism of such potentiation of the radiation effect is unknown, but most probably is cytokinetic in nature. The enhanced radiation response after fractionated pretreatment could be the result of either (1) a drug-induced redistribution or synchrony at $G_2/M$; (2) a reduced potential for the accumulation and/or repair of sublethal radiation damage; or (3) a combination of (1) and (2).

Both daily and fractionated pretreatments with ICRF-159 increased the efficacy of combined drug+radiation therapy. Whether the mechanism responsible for the enhancement is the same under both pretreatment conditions is not clear. It is likely that mechanisms other than vascular normalization are operative in short intervals after ICRF-159 administration.

This investigation was supported by Grant Number RO1 CA20328 awarded by the National Cancer Institute, DHEW.

The authors express their appreciation to Dr H. A. Hopkins for his suggestions concerning the manuscript and to Ms C. Mallick and L. Keller for their technical assistance.

### REFERENCES

Atherton, A. (1975) The effect of (±) 1,2-bis (3,5-dioxopiperazin-l,yl)propane (ICRF-159) on liver metastases from a hamster lymphoma. *Eur. J. Cancer*, **11**, 383.

Blackett, N. M. & Adams, K. (1972) Cell proliferation and the action of cytotoxic agents on haemopoietic tissue. *Br. J. Haematol.*, **23**, 751.

Denekamp, J. (1972) The relationship between the "cell loss factor" and the immediate response to radiation in animal turnovers. *Eur. J. Cancer*, **8**, 335.

Hallowes, R. C., West, D. G. & Hellmann, K. (1974) Cumulative cytostatic effect of ICRF 159. *Nature*, **247**, 487.

Hellmann, K. & Burrage, K. (1969) Control of malignant metastases by ICRF 159. *Nature*, **224**, 273.

Hellmann, K. & Field, E. O. (1970) Effect of ICRF-159 on the mammalian cell cycle: significance for its use in cancer chemotherapy. *J. Natl Cancer Inst.*, **44**, 539.

Hellmann, K. & Murkin, G. E. (1974) Synergism of ICRF 159 on radiotherapy in treatment of experimental tumors. *Cancer*, **34**, 1033.

James, S. E. & Salsbury, A. J. (1974) Effect of (±)-1,2-*bis* (3,5-dioxopiperazin-l-yl)propane on tumor blood vessels and its relationship to the antimetastatic effect in the Lewis lung carcinoma. *Cancer Res.*, **34**, 839.

Kovacs, C. J., Evans, M. J., Wakefield, J. A. & Looney, W. B. (1977) A comparative study of the response to radiation by experimental tumors with markedly different growth characteristics. *Radiat. Res.*, **72**, 455.

LeServe, A. W. & Hellmann, K. (1972) Metastases and the normalization of tumor blood vessels by ICRF-159: A new type of drug action. *Br. Med. J.*, i, 597.

Norpoth, K., Schaphaus. A. Ziegler H. & Witting V. (1974) Combined treatment of the Walker tumour with radiotherapy and ICRF-159. *Z. Krebsforsch.*, **82**, 324.

Peters L. J. (1976) Modification of the radiocurability of a syngeneic murine squamous carcinoma by its site of growth by electron-affinic drugs and by ICRF-159. *Br. J. Radiol.*, **49**, 708.

Ryall R. D. H. Harham I. W. F. Newton K. A. Hellmann, K., Brinkley D. & Hjertaas O. K. (1974) Combined treatment of soft tissue and osteosarcoma by radiation and ICRF-159. *Cancer*, **34,** 1040.

SALSBURY A. J. BURRAGE K. & HELLMANN K. (1970) Inhibition of metastatic spread by I.C.R.F. 159: Selective deletion of a malignant characteristic. *Br. Med. J.*, iv, 344.

SALSBURY, A. J., BURRAGE K. & HELLMANN K. (1974) Histological analysis of the antimetastatic effect of (±) 1 2-bis (3 5-dioxopiperazin-1-yl)-propane. *Cancer Res.*, **34,** 843.

SIMPSON-HERREN, L., SANFORD, A. H. & HOLMQUIST J. P. (1974) Cell population kinetics of transplanted and metastatic Lewis lung carcinoma. *Cell Tissue Kinet.*, **7,** 349.

SHARPE, H. B. A., FIELD, E. O. & HELLMANN, K. (1970) Mode of action of the cytostatic agent 'ICRF-159'. *Nature*, **226,** 524.

STANLEY J. A. SHIPLEY, W. U. & STEEL, G. G. (1977) Influence of tumour size on hypoxic fraction and therapeutic sensitivity of Lewis lung tumour. *Br. J. Cancer*, **36,** 105.

STEPHENS, T. C. & CREIGHTON, A. M. (1974) Mechanism of action studies with ICRF-159: Effects on the growth and morphology of BHK-215 cells. *Br. J. Cancer*, **29,** 99.

TAYLOR, I. W. & BLEEHEN, N. M. (1977*a*) Changes in sensitivity to radiation and ICRF-159 during the life of monolayer cultures of EMT6 tumour line. *Br. J. Cancer*, **35,** 587.

TAYLOR, I. W. & BLEEHEN, N. M. (1977*b*) Interaction of ICRF-159 with radiation, and its effect on sub-lethal and potentially lethal radiation damage *in vitro*. *Br. J. Cancer*, **36,** 493.